# Dynamics of career intentions in a medical student cohort: a four-year longitudinal study

**DOI:** 10.1186/s12909-023-04102-w

**Published:** 2023-02-27

**Authors:** Eva Pfarrwaller, Lionel Voirol, Mucyo Karemera, Stéphane Guerrier, Anne Baroffio

**Affiliations:** 1 University Institute for Primary Care, Faculty of Medicine, University of Geneva, Rue Michel-Servet 1, 1211 Genève 4, Geneva, Switzerland; 2 Unit of Development and Research in Medical Education, Faculty of Medicine, University of Geneva, Geneva, Switzerland; 3 Research Center for Statistics, Geneva School of Economics and Management, University of Geneva, Geneva, Switzerland; 4 Faculty of Science, University of Geneva, Geneva, Switzerland

**Keywords:** Career choice, Undergraduate medical education, Cohort study

## Abstract

**Background:**

Medical students’ career intentions often change between matriculation and graduation, yet little is known about the precise timing and dynamics of individual students’ career decisions. This study expands on previous research by exploring the stability of individual students’ career intentions over four years and by analyzing associations between unstable career intentions and students’ characteristics.

**Methods:**

Medical students from two classes were recruited into a cohort during their first academic year and completed a yearly survey over a four-year period (end of pre-clinical curriculum to graduation). Measures included career intention (specialty and practice type), personality, coping strategies, empathy, and motives for becoming a physician. The authors developed a score ranging from 0 to 10 quantifying instability of career intentions (0 = stable; 10 = unstable). The distribution of the score was analyzed descriptively, and the association between the score and other variables was quantified using a stepwise beta regression model.

**Results:**

The sample included 262 students (61% females). The mean score was 3.07 with a median of 3. 18% of students (N = 46) did not change their specialty intention over the four years, whereas 10% (N = 26) changed every year. No further subgroups were identified between these extremes. An intention to work in private practice in year 3 and the motive *care for patients* were significantly associated with more stable career intentions.

**Conclusion:**

Most students are situated on a continuum between the two extremes of being firmly committed and undecided. Extrinsic factors may be more important drivers of these fluctuations than personal characteristics and should be explored in future research. This study’s findings also provide avenues for supporting students in their career decision-making.

**Supplementary Information:**

The online version contains supplementary material available at 10.1186/s12909-023-04102-w.

## Background

Undergraduate medical students’ career choices result from a dynamic decision-making process and ultimately determine medical workforce composition [1]. Medical schools need to be aware of their responsibility to contribute to a physician workforce aligned with population health needs [2]. Profound knowledge about the process of career choice is crucial as it supports the development of undergraduate curricula that contribute to orienting students towards medical fields that are most relevant for an effective health system, such as primary care [3]. Students’ specialty choices are influenced by personal characteristics, individual career-related needs, medical schools’ characteristics, and the undergraduate curriculum [1, 4]. Yet, little is known on the timing and stability of individual students’ career decisions [5]. This paper focuses on the dynamics of undergraduate medical students’ career intentions and suggests a method to quantify career instability, aiming to inform medical schools’ initiatives related to career choice and career planning.

The study is theoretically grounded in a previously developed conceptual framework of primary care career choice, based on a broad literature in the area of career choice [6]. It was developed to visualize the longitudinal decision-making process through which students make career-related choices during undergraduate medical education. It notably included the hypothesis, drawn from another theoretical framework, that students’ career choice pathways from matriculation to graduation may be described through four distinct trajectories depending on the strength of their commitment to primary care [7]. However, the need for further research on how students’ career choices evolve over time was highlighted. Indeed, previous research suggested that students’ career intentions often change during medical school. Most findings were based on comparisons of students’ career intentions at matriculation and graduation, i.e., at two points in time [8–10]. Thus, they do not inform about the timing or stability of career intentions [5, 11]. However, we expect students to differ according to how their career intentions evolve over time and how much they are committed to a career [6, 7]. These trajectories may affect students’ reactions to medical school experiences and career planning needs [5]. Recent research has suggested that students deciding early on their future specialty actively plan their career, whereas more undecided ones tend to approach career planning more passively and may thus benefit from structured career guidance [12, 13].

From a public health perspective, it may be most interesting to better understand the students who are not strongly committed to a specialty or who are undecided, as they might be oriented toward specialties according to public health needs, such as primary care [7]. Previous studies observed that changing career preferences and career indecision were influenced by medical school experiences [14–17] and by students’ personality and coping strategies [15, 18]. The impact of other student-related factors, such as empathy, on changing career preferences in medical students has not been studied to our knowledge. Associations between the stability of career preferences and certain specialties have been observed, although findings remain inconsistent regarding which specialties were associated with more stable preferences [8, 10, 19].

Few studies analyzed career intentions longitudinally using more than two time points. We recently described career intention dynamics in a four-year cohort with a focus on primary care, observing that changes predominated in early clinical years [20]. However, this analysis could not conclude on the stability of *individual* career intentions. Additionally, we observed that students’ motives for becoming a physician were associated with primary care career preferences. Most of these motives remained stable over a four-year period, suggesting that they might be related to students’ intrinsic characteristics and thus be important factors to consider when studying the development of career choices. Other authors explored career intentions in a cohort of 24 students, developing a score quantifying career intention stability [11]. Their findings suggested three trajectories: students with stable career intentions; students considering two or three options; and students with unstable preferences. To our best knowledge, this was the first study quantifying career intention stability using more than two data points. Its findings are however limited by the small sample size.

Our present study’s aim was to expand on previous research and contribute to filling research gaps by quantifying individual instability of career intentions in a larger student cohort over four years, and by exploring its association with students’ personal characteristics. The objectives were to: (1) describe how individual students’ career intentions evolve over several years by calculating a score reflecting the instability of career intentions; and (2) study the association between individual students’ scores and their personal characteristics and motives to become physicians.

## Methods

### Study design

We analyzed longitudinal data from an undergraduate medical student cohort followed over four years. The present report is one of several from this cohort study, which started in 2011. It was designed to study the impact of student and institutional factors on academic performance and career choices over the course of the entire undergraduate curriculum. The analysis presented in this paper is based on a selection of variables considered relevant for answering the research objectives. We briefly present the study design and context; more details and other findings related to this cohort may be found in previous publications [21–24].

### Setting

The study took place at the Faculty of Medicine in Geneva, Switzerland. The six-year undergraduate curriculum consists of a pre-selection year (year 1), two pre-clinical years (years 2 and 3), two years of compulsory clinical clerkships (years 4 and 5), and one elective year (year 6). Medical graduates pursue postgraduate training in the field of their choice; change of career orientation is possible during postgraduate training.

### Participants

All students from two consecutive classes, starting medical school in 2011 and 2012, were recruited into a cohort study during their first academic year and invited to complete a yearly paper-and-pencil survey. From a total of 306 students in these two classes, 290 (95%) enrolled into the study. Participants signed a consent form after being informed about the study. They provided a unique student identification number at each data collection to allow longitudinal matching of questionnaires. Researchers involved in the analysis could not identify students through their identification numbers to guarantee confidentiality. For the present study, we considered data collected from academic year 3 (i.e., end of pre-clinical training) to year 6 (i.e., before final certification exams). We included individuals who had provided career intentions in at least three out of these four years. Our sample thus included 262 students (i.e., 85.6% of all students in these two classes).

### Student-related variables

Table 1 presents the variables chosen from the cohort dataset according to their relevancy for the objective of the present study. **Career intentions** were assessed by two single-choice questions: (1) “What type of practice do you plan to exercise in the future?” (options: private practice, hospital practice, or teaching/research), and (2) “If you are considering a specialization, which one?” (options: anesthesiology, general internal medicine, internal medicine subspecialty, emergency medicine, obstetrics-gynecology, pathology, pediatrics, psychiatry, radiology, surgery, academic activity, undecided, and other with the option of specifying a hand-written specialty). The list of specialties was derived from postgraduate specializations available in Switzerland. For the statistical analysis, specialties were grouped into the following six categories: General medicine (general internal medicine, pediatrics), medical specialties, surgical specialties (surgery, gynecology/obstetrics, ophthalmology), acute care (anesthesiology and intensive care, emergency medicine), technical specialties (radiology and medical informatics, pathology and forensic medicine), and other (psychiatry, academic activity, other specialties).

Students were asked whether they had identified a positive **role model** during their studies (answer options: yes or no); this variable was included in our analysis as we hypothesized that having a role model could be associated with more stable career plans [25].

**Motives for becoming a physician** were included because of their previously observed association with certain career choices [20]. In our previous study, we also observed that motives remained mostly stable; therefore, we only included measures from study year 3 in the present analysis. A list of motives for becoming a physician was presented to the students: academic interest, prestige, reward, private practice, saving lives, caring for patients, cure diseases, vocation, mission, and altruism. They were asked to rate the importance of each motive on a 6-point scale (i.e., “Describe how important each of these keywords is for your choice of medicine” from 1 = not important at all to 6 = very important). This set of motives was developed based on a literature review aiming at achieving a wide enough description of different typologies of motives [26–29]. Validity analyses of the set of motives and their correlation with empathy and learning approaches has been reported previously [30]. Students’ **overall motivation to become a physician** was also included as a variable, as we hypothesized that a stronger motivation could lead to a stronger commitment to a specific career choice. Students were asked to rate their overall motivation on a 6-point scale (1 = not motivated at all to 6 = very motivated).

**Empathy** was included because it is considered a key correlate of medical students’ career choices [31, 32], emotional intelligence [33, 34], and psychological distress [35, 36], thus potentially contributing to changing career preferences. Empathy was measured using the student’s version of the Jefferson Scale of Empathy (JSE-S), consisting of a 20-item questionnaire assessing students’ perception of the importance of empathy in the doctor-patient relationship (Cronbach’s Alpha α = 0.83) [37]. Answers to questions are structured on a 7-point scale; a total score is calculated by summing up all answers. We used a French version of the JSE-S whose validity and cross-national generalizability has been confirmed [38].

**Personality** traits were included because of their associations with career indecision [39]. Also, personality has been hypothesized to play a role in changing career preferences [15]. The validated French version of the NEO Five Factor Inventory (NEO-FFI) was used [40]. It is the short version of the classic revised NEO Personality Inventory, which is the assessment instrument most often used to measure personality with the “Big Five” model, assuming five underlying personality dimensions. NEO-FFI includes 12 items for each dimension scored on a 5-point scale: neuroticism (α = 0.87), extraversion (α = 0.74), openness (α = 0.69), conscientiousness (α = 0.86), and agreeableness (α = 0.73).

**Coping strategies** were included because they have been described as important influences on career decision-making in medical students; notably, maladaptive coping strategies were associated with career indecision [18]. Career decision-making may be regarded as a potential stressor and approached differently depending on students’ strategies coping with this stress. Coping styles were measured by the Coping Inventory for Stressful Situations (CISS), which assesses coping strategies that individuals might use when facing stressful situations [41, 42]. Three dimensions of 16 items each are rated on a 5-point scale: emotional coping (α = 0.87), task coping (α = 0.89), and avoidant coping (α = 0.80). This questionnaire is widely used in the domain of psychology and has been validated in a number of languages, including French.

**Table 1:** Variables used to analyze the association between instability of career intentions and students’ characteristics

	Year of undergraduate medical studies			
	3	4	5	6
Socio-demographics: Age and gender	x			
Career intentions: • Specialty • Practice type	x	x	x	x
Having identified a positive role model				x
Motives for becoming a physician	x			
Overall motivation for becoming a physician	x			
Personality (NEO-FFI)	x			
Empathy (JSE-S)		x		
Coping strategies (CISS)		x		

Measured variables are listed in first column. « x » indicates the year of undergraduate medical studies during which the variable was measured (not all variables were measured yearly)

### Score of career intention instability

We developed a score quantifying the instability of career intentions. It was calculated for each student and composed of three elements, detailed in Table 2: (1) change of practice type intentions over the four years; (2) change of specialty intentions from one year to another; and (3) the number of different specialties indicated over the four years. We also accounted for certain specialties being closer to one another by applying a corrective factor. Career intention changes occurring later were attributed more weight as we considered these to potentially reflect a more pronounced career indecision. The score ranged from 0 (= maximum stability, i.e., career intentions never changed) to 10 (= maximum instability of career intentions). Seven students indicated being undecided in year 6 and in at least two of the three previous years; we manually attributed them the maximum score of 10 as we considered them to be highly undecided.

**Table 2:** Calculation of a score to quantify instability in career intentions in a medical student cohort

Sub-scores			Total score
	Method of calculation	Sub-score characteristics	
Practice type	Practice type intentions over the 4 years^a^: • Always the same type of practice: 0 points • Undecided + one type of practice: 0.5 point • More than one type of practice: 1 point	**Practice type sub-score** Minimum: 0 Maximum: 1 Practice type intention is accorded a small relative weight in the total score, as it is considered to play a minor role in overall career choices.	**Total score** ^e^ Sum of practice type sub-score plus specialty sub-score. Minimum: 0 Maximum: 10 A score of 10 points was manually applied to individuals having indicated being undecided about specialty in year 6 and in at least two out of the other three years.
Specialty	a. For each of the three transitions between years (from year 3 to 4, from year 4 to 5, and from year 5 to 6), changes in specialty intentions are quantified as follows^b^: i. Change of specialty intention from one year to another, attributing more weight to later years in the curriculum^c^. ii. These points are multiplied with a corrective factor to account for the similarity between the two specialties concerned by the change^d^. The points of each transition are then summed (i.e., a total of 0 to 6 points).	**Specialty sub-score** Sum of component (a) (change of intentions) and (b) (number of specialties). Minimum: 0 Maximum: 9 Greater relative weight is attributed to changes in later years and to changes between specialties not considered to be in the same category.	
	b. Number of different specialties over the 4 years^a^: • Always the same specialty: 0 points • 2 specialties: 1 point • 3 specialties: 2 points • 4 specialties: 3 points The option *undecided* was ignored in this part of the calculation.		

The score was calculated for each student in the cohort, based on career intentions collected over four consecutive years (years 3 to 6 of undergraduate medical education). Students having provided at least 3 out of 4 possible data points were included in this study. The score is composed of two sub-scores (practice type and specialty) and based on the principle that more changes in career intentions are associated with a higher score

^a^Missing data management: No imputation of missing data (sub-score based on 3 or 4 data points as available)

^b^Missing data management: For individuals with a missing data point (N = 49), the calculations were replaced by the median of the sub-score (a) in the remaining population

^c^From year 3 to 4: 1 point; from year 4 to 5: 2 points; from year 5 to 6: 3 points; no change: 0 points

^d^The decision about the “similarity” between specialties was based on discussions within the research team and on experts’ opinion. Corrective factors were applied according to the two specialties concerned by the change as follows:

• General internal medicine / Pediatrics: 0.25

• General internal medicine / Emergency medicine: 0.5

• Pediatrics / Emergency medicine: 0.5

• Medical specialties / Emergency medicine: 0.5

• Medical specialties / General internal medicine: 0.5

• Medical specialties / Pediatrics: 0.5

• Emergency medicine / Anesthesiology and intensive care: 0.5

• Surgery / Gynecology and obstetrics: 0.5

• Pathology and forensic medicine / Radiology and medical informatics: 0.5

• Ophthalmology / Medical specialties: 0.5

• Undecided / All specialties: 0.5

• All other changes: 1

^e^Calculation of the score expressed as a formula: [Total score] = [Practice type subscore] + [(Change_years 3−4_ x Correction_years 3−4_ + Change_years 4−5_ x Correction_years 4−5_ + Change_years 5−6_ x Correction_years 5−6_) + (Number of specialties)]

### Analyses

We examined data for accuracy and missingness and calculated descriptive statistics for age (mean and standard deviation) and gender (N and % of females). For an overview of our cohorts’ characteristics, we cross-sectionally described career intentions (N and % per academic year) and overall number of changes in career intentions and the respective specialties. The career intention instability score was analyzed descriptively (mean, standard deviation, median, quartiles).

We used a regression analysis to search for associations between the score (the dependent variable) and the other variables, selected because of their potential impact on career-related decision-making (see above). Specialties were grouped into six categories to limit the number of variables. Given the score’s distribution, we applied a beta regression considering the scaled instability score as the dependent variable. Moreover, considering the large number of covariates, we selected the model based on a stepwise procedure using the Generalized Akaike Information Criterion [43]. This stepwise regression method is a standard approach to select relevant covariates and to obtain an interpretable model in the presence of a large number of covariates compared to the number of observations [44, 45]. The adequacy of the model with the data was assessed with a residual analysis based on the randomized quantile residuals [46]. The model’s estimated coefficients were interpreted in terms of their magnitude (absolute value), sign (positive or negative), and *p*-value.

All analyses were carried out with R statistical software [47] and the package GAMLSS [48]. *P*-values smaller than 0.05 were considered statistically significant.

## Results

### Study population

Our sample included 262 students. The mean age in year 3 was 22.96 years (standard deviation: 3.52 years), and 61% were female. Cross-sectional specialty and practice type intentions are described in Additional file 1. The most frequent specialty intention in year 6 was general internal medicine (24.8% of the population), which was also the specialty with the highest increase compared to year 3. Surgery was the specialty with the most important decrease in students’ intentions (from 14.1% in year 3 to 8.8% in year 6). The proportion of undecided students decreased over time. These changes were most notable between years 4 and 5. The increase in the proportion of students intending to practice general internal medicine was mostly due to “gaining” undecided students, whereas the decrease in the proportion of students intending to practice surgery was mostly due to “loosing” students to medical specialties (data not shown). The detailed analysis of specialty intention changes from one year to another (Additional file 1) revealed that specialty intentions tended to become more stable over time, with fewer changes between years 5 and 6 relative to the years before.

**Fig. 1:**
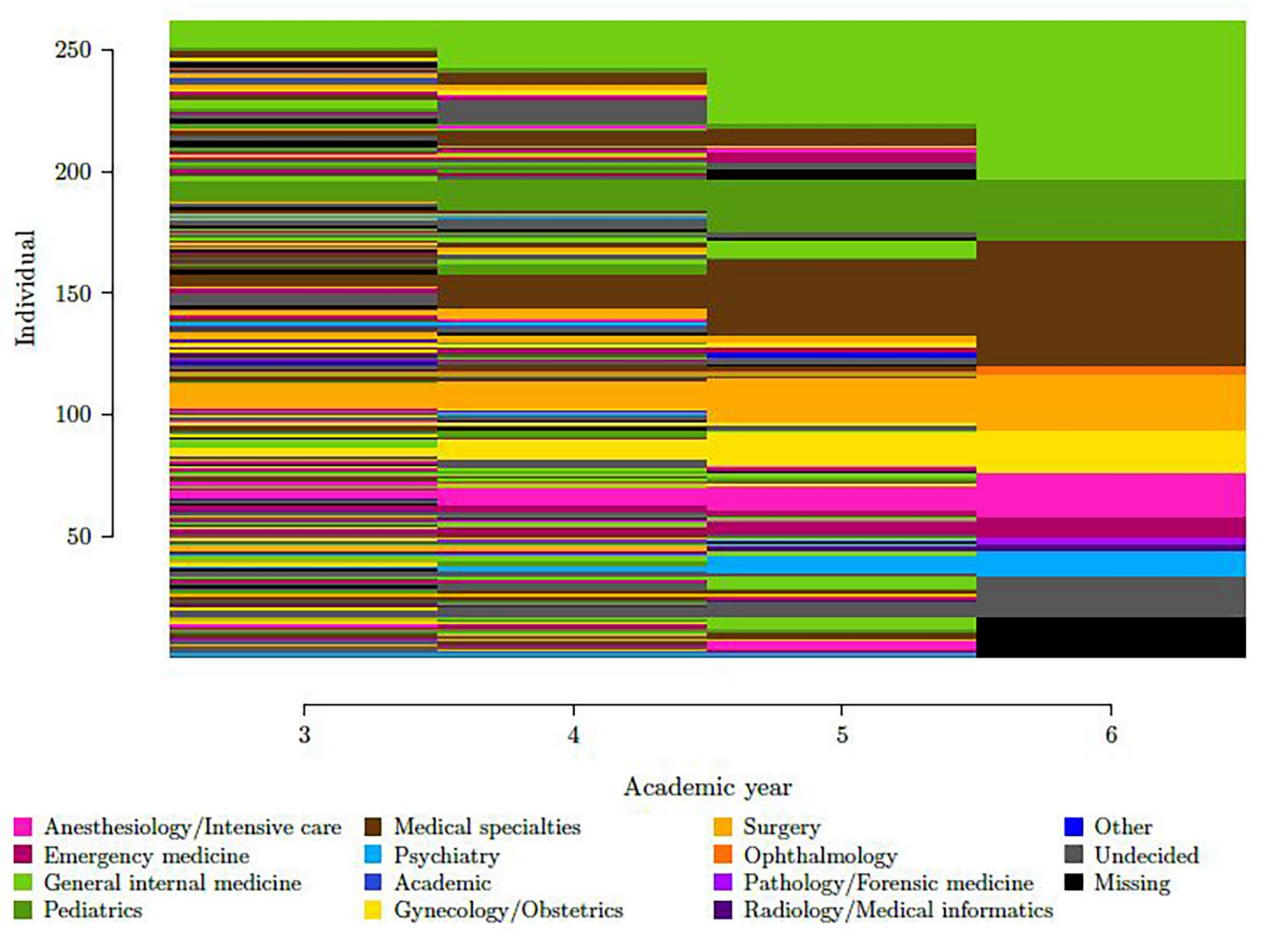
Trajectories of specialty intention in 262 medical students in a cohort followed over four years. Each student’s indicated specialty intentions over the four years are represented on one line; specialties are represented by colors (see color legend)

Figure 1 shows how specialty intentions evolved over the four years in each student (this visualization does not consider practice type intentions). 18% of students (N = 46) indicated the same specialty intention over the four years. At the other end of the spectrum, 10% of students (N = 26) changed specialty intention every year.

### Score of career intention instability

**Fig. 2:**
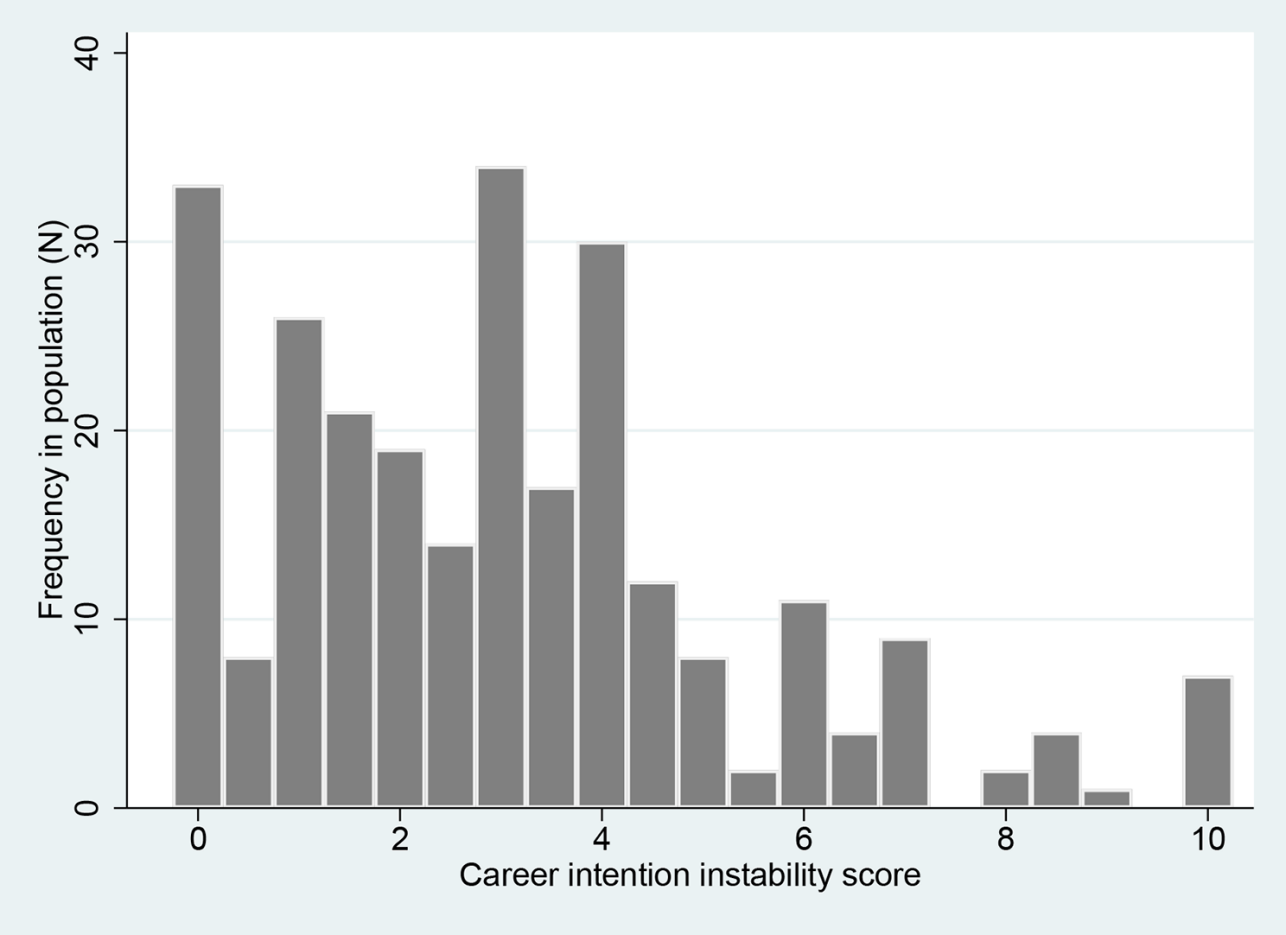
Distribution of the score of career intention instability in a cohort of 262 medical students. The score ranges from 0 (= maximal stability, i.e., no changes in career intentions over four years) to 10 (= maximal instability or students indicating being undecided in at least three years out of four)

Figure 2 shows the distribution of the score in our population. The scores ranged from 0 (maximal stability of career intentions) to 9; the seven students with a score of 10 had indicated being undecided in at least three of the four years. Thirty-three students (12.6%) had a score of 0, meaning that they indicated the same specialty and practice type choice over the four years. The distribution of the score was skewed to the left, with a mean of 3.07 (standard deviation: 2.37) and a median of 3 (first quartile: 1; third quartile: 4).

### Association between the score and other variables

The regression results of the final estimated model are presented in Table 3, considering the score as the dependent variable. Given the number of variables (see Table 1) in relation to the number of observations, we applied a stepwise variable selection method to obtain an interpretable model with reliable estimated coefficients. Hence, although all the variables presented in Table 1 were included in the statistical procedure, the variable selection method resulted in a final model containing only four variables (Table 3). A full correlation matrix including all numerical variables is available in Additional file 2. Among the four variables retained by the model, only the intention to work in private practice in year 3 and being motivated by caring for patients were significantly associated with a lower score, i.e., with more stable career intentions. Other personal characteristics, such as gender, personality, coping with stress, empathy, other motives, and specific specialty intentions were not associated with the score.

**Table 3:** Association between the score of career intention instability and other variables: Regression results of the final estimated model resulting from a stepwise variable selection procedure

Variables	Estimated coefficients^a^	Standard error	t-value	p-value^b^
(Intercept)	1.010	0.538	1.878	0.062
Motive Altruism (year 3)	0.179	0.096	1.875	0.062
Motive Cure diseases (year 3)	-0.178	0.099	-1.811	0.072
Motive Caring for patients (year 3)	**-0.279**	**0.124**	**-2.244**	**0.026**
Intention to work in private practice (year 3)	**-0.745**	**0.171**	**-4.344**	**< 0.001**

All variables presented in Table 1 were included in the statistical procedure. The stepwise variable selection procedure resulted in the final model presented in this Table, including only few variables

^a^Coefficients obtained through beta-regression model. Negative prefixes indicate an association with a lower score, i.e., with more stability in career intentions; a positive prefix indicates an association with more instable intentions

^b^P-values < 0.05 were considered statistically significant. The corresponding variables are highlighted in bold print

## Discussion

### Summary of main findings

We explored how career intentions evolved over four years in a cohort of 262 medical students and quantified the instability of career intentions through a self-developed score. A small group of students demonstrated very stable career intentions and may thus be considered highly committed to their career choice. However, some degree of instability was observed in most students. In our sample, the score was neither associated with personal characteristics such as personality, empathy, or coping strategies, nor with a specific specialty preference. Only the intention to work in private practice in the last pre-clinical year was clearly associated with more stable career intentions, and to a lesser degree, the motive “caring for patients”.

### Interpretation and comparison with the literature

Previous studies have mostly explored the stability of career choices using only two measures (typically, at entry and exit of medical school) [8–10]. Our findings confirm that career intentions are subject to change in most students and expand on previous findings by describing the dynamics of career intentions longitudinally. Career intentions became more stable in the final year of medical school, matching previous findings suggesting that clinical clerkships contribute substantially to students’ career decisions [1, 15, 16]. In our context, this means that the compulsory clinical clerkships in the main specialties in years 4 and 5 may be important in stabilizing career choices, whereas the final year (composed of mostly freely chosen clerkships) may mostly serve to confirm these career intentions.

The conceptual basis used for our study suggested that career intention paths were based on the strength of commitment to a specific career option [6]. Our score supports elements of these hypothesized trajectories by identifying subsets of strongly committed students with very stable intentions and undecided students with unstable intentions. These findings show parallels with those from the study conducted by Querido and colleagues, expanding them to a much larger cohort [11]. However, while they identified three subgroups in their small student cohort based on career intention stability, the “intermediate” group was not as clear cut in our study: our analysis rather identified a continuum of career intention stability between the extremes of being very stable and very unstable or undecided.

These findings are worth linking to another recent qualitative study [12] that suggested two main patterns of career decision-making: students strongly committed to a career used efficient career planning strategies, whereas more undecided or hesitant students demonstrated a variety of behaviors, including active (such as exploring options) and passive ones (such as letting things unfold naturally or waiting for opportunities to emerge). Considering these insights in the interpretation of our findings, we hypothesize that students situated on the continuum mentioned above may be a heterogeneous group applying multiple career decision-making strategies.

Our study also expands on previous research by exploring associations between unstable career intentions and other student-related variables. Students intending to work in private practice (i.e., ambulatory and extra-hospital practice) at the end of pre-clinical education showed a more stable career intention path, meaning that they could have had a firmer idea about their professional future early on. Students motivated by *caring for patients* also had more stable career intentions. Instability of career intentions was not associated with gender, personality, empathy, or coping strategies, suggesting that fluctuations in career intentions may be more strongly influenced by extrinsic factors. The framework used as our theoretical basis suggests that students’ experiences during medical school may be important drivers of the transformation of broad professional interest profiles into firm career intentions. Moreover, changes in career intentions may result from other factors not measured in our study, such as psychological distress, which has been suggested to foster career indecision [18].

### Strengths and limitations

Our study is based on a theoretical framework of medical student career choice [6] and draws inspiration from and expands on a previously published study quantifying career intention stability [11]. To our knowledge, this is the first study using a large cohort to describe career intention dynamics using more than two time points, to quantify career intention indecision, and to test associations with other variables. Thus, our findings provide unique insights into the dynamics of career decisions.

Our study is limited by its quantitative nature and the restricted number of survey questions related to career choice; also, students were not asked about the strength of commitment to a career. Our study was not designed to suggest reasons for evolving career intentions; however, comparing our findings with recent qualitative studies has suggested possible interpretations. Further qualitative research will need to explore the dynamics of career intentions in more detail to allow for further interpretation of our findings.

Our study is further limited by its focus on a single medical school; the findings thus need to be interpreted considering the context, especially regarding the liberal postgraduate training system in Switzerland. This context may explain why many students turned to general internal medicine (a broad specialty often viewed as a basis for further specialization). Thus, our findings may only be generalizable to contexts with a similar liberal system.

### Implications for research

Future research exploring the dynamics of the career choice process may fill further gaps, notably regarding the large group of students whose career preferences fluctuate. Ultimately, different underlying aspects may appear as unstable career intentions, such as exploratory behaviors as a normal part of career decision-making [12], a lack of focused professional interests, or career indecision with difficulties committing to a choice [49]. Future research should focus on exploring the reasons behind the varying degrees of career intention fluctuation and its meanings for career choice. Additionally, investigating students’ perceptions of this process may add further insights; some students may be comfortable with leaving things open, whereas others may feel at a loss. Qualitative methodologies are especially suited to answering these kinds of questions; a longitudinal approach would be particularly beneficial when exploring the career choice process.

### Implications for practice

The varying degrees of career intention instability may translate into different needs regarding career choice and planning support, with implications for career counseling. Undecided students may benefit from structured career planning support to better define their interests, clarify available options, and find effective ways to explore them. Committed students may need guidance on how to confirm their interests and plan their careers. Professionals offering career guidance in medical school should thus be aware of this heterogeneity in career decision-making and find out where individual students are positioned in this process (e.g., being strongly committed, exploring possible options, letting things evolve as opportunities arise, or being undecided about the future).

The insights into career decision-making also have potential implications for promoting certain career choices, especially specialties struggling with recruitment. Undecided or weakly committed students may still be malleable regarding career choices and are thus potentially “recruitable” to these specialties. These students may strongly rely on opportunities encountered during undergraduate education to make career decisions without actively exploring options. Creating diverse opportunities in the formal curriculum could thus enlarge the scope of career possibilities for some students. For others, a wide offer of experiences may favor exploration of career options and thus contribute to settling career decisions according to their interests and needs. We strongly suggest that the development, implementation, and evaluation of initiatives to support students’ career decision-making be based on our and previous studies’ findings as well as available theoretical concepts about career choice.

## Conclusion

The score developed in our longitudinal study suggests that medical students’ career intentions change to varying degrees, with most students situated on a continuum between being firmly committed and fully undecided. Our findings provide avenues for future research, which should explore factors that contribute to students changing career intentions, and the strategies used in their decision-making processes. This study’s insights expand on previous findings and may inform initiatives aiming to support students in their career choice process; they also provide further ideas about how to recruit young physicians to careers that are dealing with workforce shortages.

## Electronic supplementary material

Below is the link to the electronic supplementary material.

Supplementary Material 1

Supplementary Material 2

## Data Availability

The datasets analyzed for the study presented here are available from the corresponding author on reasonable request.
