# Supplementary material for: Dynamics of career intentions in a medical student cohort: a four-year longitudinal study
Source: BMC Med Educ. 2023 Feb 27;23:131. doi: 10.1186/s12909-023-04102-w (PMC9972700; doi:10.1186/s12909-023-04102-w)
Supplement: Supplementary file 1 — Supplementary Material 1 [file 12909_2023_4102_MOESM1_ESM.docx]

**Additional file 1: Cross-sectional description of specialty intentions and changes of specialty intentions in a cohort of 262 medical students**

**2.1: Specialty and practice type intentions in the cohort, by academic year**

Data are presented as numbers and percentage of total population (i.e., 262).

|  | Year 3 | Year 4 | Year 5 | Year 6 |
| --- | --- | --- | --- | --- |
| Specialty intentions |  |  |  |  |
| General internal medicine | 33 (12.6%) | 42 (16%) | 69 (26.3%) | 65 (24.8%) |
| Pediatrics | 22 (8.4%) | 34 (13%) | 28 (10.7%) | 25 (9.5%) |
| Medical specialties | 34 (13%) | 41 (15.6%) | 46 (17.6%) | 52 (19.8%) |
| Surgery | 37 (14.1%) | 33 (12.6%) | 24 (9.2%) | 23 (8.8%) |
| Gynecology/Obstetrics | 17 (6.5%) | 15 (5.7%) | 19 (7.3%) | 18 (6.9%) |
| Ophthalmology | 1 (0.4%) | 1 (0.4%) | 1 (0.4%) | 3 (1.1%) |
| Anesthesiology/Intensive care | 8 (3.1%) | 11 (4.2%) | 17 (6.5%) | 18 (6.9%) |
| Emergency medicine | 23 (8.8%) | 20 (7.6%) | 17 (6.5%) | 8 (3.1%) |
| Pathology/Legal medicine | 1 (0.4%) | 1 (0.4%) | 0 (0%) | 3 (1.1%) |
| Radiology/Medical informatics | 6 (2.3%) | 2 (0.8%) | 3 (1.1%) | 3 (1.1%) |
| Psychiatry | 5 (1.9%) | 7 (2.5%) | 8 (3.1%) | 10 (3.8%) |
| Other specialties/Academic activity | 3 (1.1%) | 1 (0.4%) | 3 (1.1%) | 0 (0%) |
| Undecided | 52 (19.8%) | 49 (18.7%) | 20 (7.6%) | 17 (6.5%) |
| Missing | 20 (7.6%) | 5 (1.9%) | 7 (2.5%) | 17 (6.5%) |
| Practice type intentions |  |  |  |  |
| Private practice | 69 (26.3%) | 87 (33.2%) | 106 (40.5%) | 108 (41.2%) |
| Hospital practice | 122 (46.6%) | 119 (45.4%) | 112 (42.7%) | 89 (34%) |
| Other practice type | 8 (3.1%) | 11 (4.2%) | 5 (1.9%) | 6 (2.3%) |
| Undecided | 43 (16.4%) | 41 (15.6%) | 33 (12.6%) | 42 (16%) |
| Missing | 20 (7.6%) | 4 (1.5%) | 6 (2.3%) | 17 (6.5%) |

**2.2: Description of changes in specialty intentions in the cohort, from one academic year to another**

|  | From year 3 to 4 | From year 4 to 5 | From year 5 to 6 |
| --- | --- | --- | --- |
| Specialty intentions: |  |  |  |
| Stable (same specialty intention in both years) N (% of total population) | 125 (48%) | 119 (45%) | 158 (60%) |
| Change (change in specialty intention from one year to another) N (% of total population) | 112 (43%) | 131 (50%) | 80 (31%) |
| N/A^a^ N (% of total population) | 25 (9%) | 12 (5%) | 24 (9%) |
| Three most frequent types of changes (N) | Undecided / Medical specialties (18)  Undecided / General internal medicine (11)  General internal medicine / Medical specialties (7) | Undecided / General internal medicine (15)  General internal medicine / Medical specialties (12)  Undecided / Pediatrics (10) | General internal medicine / Medical specialties (14)  Undecided / General internal medicine (8)  General internal medicine / Emergency medicine (5) |
| Three most frequent stable specialties^b^ (N) | Surgery (24)  General internal medicine (22)  Pediatrics (16) | General internal medicine (26)  Medical specialties (23)  Surgery (17) | General internal medicine (42)  Medical specialties (31)  Pediatrics (22) |

^a^Due to missing survey data in one of the two years.

^b^Not including undecided.
