# Supplementary figures and images for: Dynamics of career intentions in a medical student cohort: a four-year longitudinal study
Source: BMC Med Educ. 2023 Feb 27;23:131. doi: 10.1186/s12909-023-04102-w (PMC9972700; doi:10.1186/s12909-023-04102-w)

Additional file 2: Full correlation matrix of numerical variables

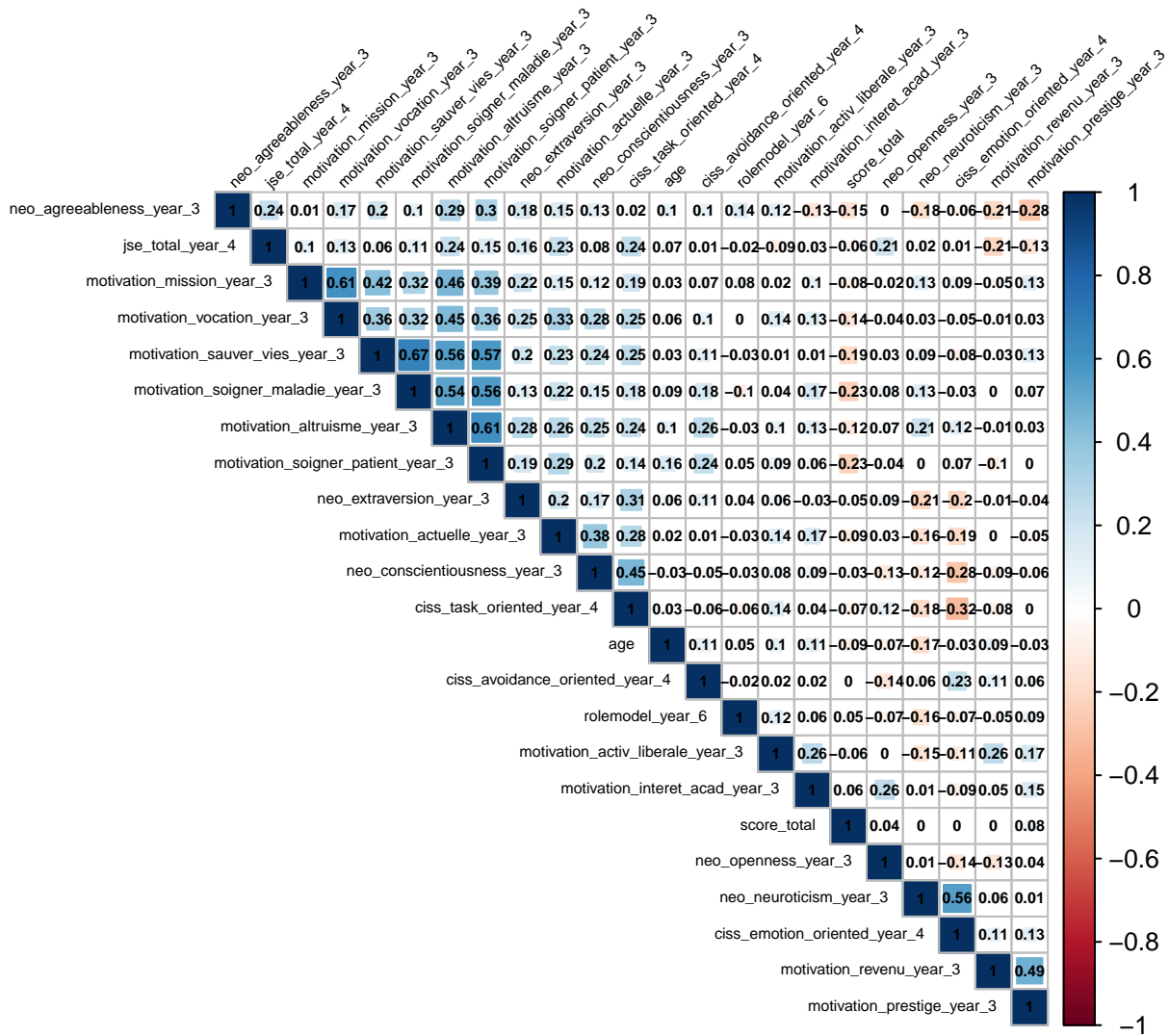

Supplement: Supplementary file 2 — Supplementary Material 2 [file 12909_2023_4102_MOESM2_ESM.pdf]
